# Mitochondrial Mechanisms in Septic Cardiomyopathy

**DOI:** 10.3390/ijms160817763

**Published:** 2015-08-03

**Authors:** María Cecilia Cimolai, Silvia Alvarez, Christoph Bode, Heiko Bugger

**Affiliations:** 1 Department of Cardiology and Angiology, Heart Center Freiburg University, Hugstetter Str. 55, 79106 Freiburg, Germany; E-Mails: cecilia.cimolai@universitaets-herzzentrum.de (M.C.C.); christoph.bode@universitaets-herzzentrum.de (C.B.); 2 Institute of Biochemistry and Molecular Medicine, School of Pharmacy and Biochemistry, University of Buenos Aires-National Scientific and Technical Research Council (UBA-CONICET), Junín 956, C1113AAD Buenos Aires, Argentina; E-Mail: salvarez@ffyb.uba.ar

**Keywords:** septic cardiomyopathy, mitochondrial dysfunction, heart, bioenergetics

## Abstract

Sepsis is the manifestation of the immune and inflammatory response to infection that may ultimately result in multi organ failure. Despite the therapeutic strategies that have been used up to now, sepsis and septic shock remain a leading cause of death in critically ill patients. Myocardial dysfunction is a well-described complication of severe sepsis, also referred to as septic cardiomyopathy, which may progress to right and left ventricular pump failure. Many substances and mechanisms seem to be involved in myocardial dysfunction in sepsis, including toxins, cytokines, nitric oxide, complement activation, apoptosis and energy metabolic derangements. Nevertheless, the precise underlying molecular mechanisms as well as their significance in the pathogenesis of septic cardiomyopathy remain incompletely understood. A well-investigated abnormality in septic cardiomyopathy is mitochondrial dysfunction, which likely contributes to cardiac dysfunction by causing myocardial energy depletion. A number of mechanisms have been proposed to cause mitochondrial dysfunction in septic cardiomyopathy, although it remains controversially discussed whether some mechanisms impair mitochondrial function or serve to restore mitochondrial function. The purpose of this review is to discuss mitochondrial mechanisms that may causally contribute to mitochondrial dysfunction and/or may represent adaptive responses to mitochondrial dysfunction in septic cardiomyopathy.

## 1. Introduction

Sepsis is responsible for millions of deaths worldwide each year and is a frequent cause of death in people who have been hospitalized [1]. In the United States, 3 in 1000 people suffer from sepsis, and severe sepsis contributes to more than 200,000 deaths per year [2]. Sepsis is the manifestation of the immune and inflammatory response to infection that may ultimately result in multi organ failure. It is believed that the release of pro-inflammatory mediators is not answered by an appropriate anti-inflammatory response and overwhelms the immune system, resulting in an uncontrolled excessive inflammatory state and an inability to neutralize pathogens [3]. Both effects of bacterial endotoxins and exotoxins, as well as an excessive release of a large number of cytokines contribute to organ dysfunction and failure during sepsis and septic shock. Guidelines recommend early treatment with antibiotics, to restore fluid deficits, and vasopressor treatment if necessary, actions that are critical for patient outcomes and overall survival. Besides specifically neutralizing the pathogen with antibiotics, the currently recommended therapeutic strategies are symptomatic, and no specific treatment is available to treat the consequences of specific organ damage in sepsis.

End-organ damage and organ failure in sepsis affects the most significant organs of the body, including the heart. Myocardial dysfunction is a well-described complication of severe sepsis, also referred to as septic cardiomyopathy, which includes both systolic and diastolic dysfunction [4]. Adequate O_2_ supply in sepsis suggests that myocardial depression is not related to tissue hypoperfusion but to the presence of circulating depressant factors, or other mechanisms [5]. A number of mechanisms have been proposed to be involved in myocardial dysfunction in sepsis, including toxins, cytokines, nitric oxide, complement activation, apoptosis and energy metabolic derangements [6,7]. The latter two mechanisms imply that mitochondrial function may be compromised in septic cardiomyopathy. Already a century ago, it had been recognized that low arterial oxygen tension, low circulating hemoglobin levels and/or tissue hypoperfusion may lead to tissue hypoxia during sepsis, thereby contributing to impaired ATP generation [8,9,10]. Others demonstrated that these parameters were not impaired or even improved during sepsis [11,12]. Instead, more recent studies strongly suggest that organ dysfunction in sepsis, including myocardial depression, is rather related to the presence of circulating depressant factors or other mechanisms, leading to cellular energy depletion [5].

Indeed, a number of studies identified myocardial mitochondrial dysfunction in septic conditions both in animal models and humans [13,14,15,16,17,18]. Since the heart is highly dependent on continuous delivery of ATP to maintain contractile function, impairment in mitochondrial function is energetically detrimental for the heart. For a number of cardiac pathologies, mitochondrial dysfunction and energy depletion have been proposed to significantly contribute to cardiac dysfunction, including prevalent cardiac diseases such as ischemia reperfusion injury, diabetic cardiomyopathy and heart failure [19,20,21]. While a number of mechanisms have been proposed to causally contribute to myocardial mitochondrial dysfunction in sepsis, a number of other mitochondria-related alterations occur in septic cardiomyopathy the purpose of which may be to restore mitochondrial function. In the following sections, we will review the evidence of mitochondrial dysfunction in septic cardiomyopathy and discuss mitochondrial mechanisms that may causally contribute to mitochondrial dysfunction and/or may represent adaptive responses to mitochondrial dysfunction in septic cardiomyopathy.

## 2. Myocardial Mitochondrial Dysfunction in Sepsis

In the heart, high-energy phosphates are mainly generated from the oxidation of fatty acids, while glucose and other substrates contribute to myocardial ATP regeneration to a lesser extent. Substrate oxidation yields mainly NADH (reduced nicotinamide adenine dinucleotide) and FADH_2_ (reduced flavin adenine dinucleotide), which feed electrons into the electron transport chain of oxidative phosphorylation (OXPHOS) via complex I and II. Electron transport results in the reduction of O_2_ to H_2_O, which is coupled to ATP regeneration by the F_0_F_1_-ATPase (ATP synthase). ATP exits the mitochondrion and is mainly utilized by myosin ATPases to maintain contractile function, whereas a minor part of ATP is used to maintain cellular ion homeostasis. Of note, the entire pool of myocardial ATP is turned over completely every few seconds, emphasizing that mitochondrial energy substrate metabolism is critically important to maintain ATP availability and thus cardiac function.

Mitochondrial dysfunction is widely discussed as a crucial mechanism of organ dysfunction in sepsis, including the heart, although only few data on mitochondrial function are available from human septic hearts. Takasu and colleagues reported edema of the mitochondrial matrix, associated with cystic alterations of the cristae and collapse into small myelin-like clusters in hearts of septic patients [22]. Soriano *et al.* observed that patients that did not survive sepsis presented a more severe degree of cardiac dysfunction compared to survivors, and non-survivors also showed derangements in mitochondrial cristae [13]. In contrast, numerous animal studies reported myocardial mitochondrial dysfunction in sepsis. The most commonly used animal models of sepsis are the induction of sepsis by injection of an exogenous bacterial toxin, mostly lipopolysaccharide (LPS), or the induction of intestinal leakage by cecal ligation and puncture (CLP) or colon ascendant stent peritonitis (CASP) [23]. Using these models, it was well documented by Smeding and colleagues [24] that, in the majority of the cases, the impairment in cardiac mitochondrial function correlated with decreased cardiac contractility, both measured *in vivo* by echochardiography or *ex vivo* in the isolated heart perfusion. Mitochondrial dysfunction was characterized by decreased rates of State 3 respiration and ATP synthesis, decreased respiratory control ratios and membrane potential, decreased activities of mitochondrial OXPHOS Complexes, increased rates of State 4 respiration, and increased mitochondrial size and fragility [14,25,26,27,28,29]. Several authors reported a decrease in State 3 respiration in the heart using glutamate as a Complex I substrate, whereas an impairment in Complex II-mediated respiration was less consistent [29,30,31]. Decreased activity or expression of individual OXPHOS Complexes was also widely reported, in particular Complex I. Decreased State 3 respiration was frequently associated with a decreased respiratory control ratio (RCR), although the lower RCR was sometimes related to increased State 4 respiration [26], which could be indicative of mitochondrial uncoupling. In addition, a decrease of the mitochondrial transmembrane potential (Δψ) or increased mitochondrial permeability transition was often observed in animal models of sepsis. Functional impairment of cardiac mitochondria was often associated with structural defects of mitochondria, such as cristae derangement, cleared matrix and mitochondrial swelling in animal models, thereby confirming the findings in human septic hearts as described above. Finally, increased mitochondrial reactive oxygen species (ROS) generation also confirms dysfunction of mitochondria in septic cardiomyopathy [32].

**Figure 1:**
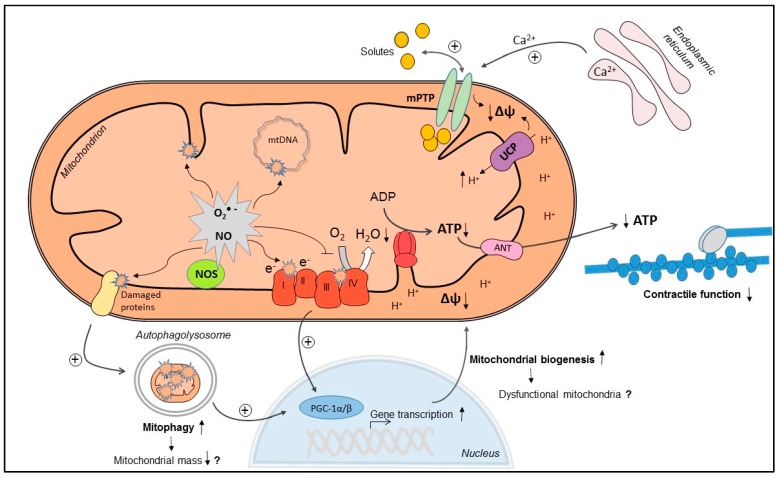
Mitochondrial mechanisms in septic cardiomyopathy: Increased superoxide (O_2_·**^−^**) and nitric oxide (NO) production can cause direct oxidative or nitrosative damage and inhibition of oxidative phosphorylation (OXPHOS) complexes, leading to decreased O_2_ consumption and decreased mitochondrial membrane potential (Δψ). In addition, Δψ may drop due to increased uncoupling protein (UCP)-mediated proton leak, increased Ca^2+^-induced mitochondrial permeability transition pore (mPTP) opening and direct oxidative damage of the inner mitochondrial membrane. As a consequence, mitochondrial ATP regeneration is compromised, and energy depletion may contribute to cardiac contractile dysfunction. Increased mitophagy may eliminate dysfunctional mitochondria, which may be replaced by increased mitochondrial biogenesis, mediated by activation of peroxisome proliferator-activated receptor γ coactivator 1α/β (PGC-1α/β). However, if uncoordinatedly activated, mitophagy and mitochondrial biogenesis may lead to decreased mitochondrial mass and dysfunctional mitochondria (upward pointing arrow: increase; downward pointing arrow: decrease; dashed arrow: possible consequence).

Although mitochondrial dysfunction in septic cardiomyopathy is associated with cardiac dysfunction, the underlying mechanisms of mitochondrial dysfunction are still incompletely elucidated. In addition, a number of mitochondrial mechanisms are triggered in septic cardiomyopathy, which may rather serve to restore than impair mitochondrial function, although controversially discussed. In the following sections, we will discuss mitochondrial mechanisms that may causally contribute to mitochondrial dysfunction and/or may represent adaptive responses to mitochondrial dysfunction in septic cardiomyopathy (illustrated in Figure 1).

## 3. Mechanisms of Myocardial Mitochondrial Dysfunction in Sepsis

### 3.1. Mitochondrial NO Production and Oxidative Stress

During respiration, mitochondria may reduce O_2_ univalently thereby producing O_2_·^−^ (superoxide anion) as a normal metabolite. Under physiological conditions, only a small percentage (1%–2%) of the O_2_ utilized is driven to O_2_·^−^ formation, which is dismutated to H_2_O_2_ by Mn-superoxide dismutase. However, situations in which steady-state concentrations of these reactive oxygen species (ROS) are increased can lead to a diverse array of reversible and irreversible toxic modifications on biomolecules, such as protein carbonylation or lipid peroxidation. The continuous mitochondrial production of free radicals and the significant amount of mitochondria within cells makes mitochondria the most important source of these harmful species within the cell. At the same time, mitochondrial enzymes, function and mtDNA are particularly sensitive to ROS-induced damage [33,34]. Mitochondria also produce nitric oxide (NO) through the activity of mitochondrial NO synthase (mtNOS), which physiologically regulates mitochondrial respiration by inhibition of cytochrome c oxidase [35].

Sepsis is an excessive, systemic inflammatory response characterized by massive increases in ROS, NO and inflammatory cytokines [36]. A large body of evidence strongly suggests that excessive mitochondrial production of NO and ROS contribute to mitochondrial dysfunction in sepsis in various tissues, including the heart. Studies demonstrated that NO production, production of O_2_·^−^ and H_2_O_2_, global protein nitration, nitrotyrosine content, protein carbonylation and lipid peroxidation are increased in cardiac mitochondria of animals subjected to endotoxemia [14,26,37,38,39]. Moreover, the antioxidant systems, aimed to scavenge these reactive species, seem to be inhibited as shown by decreased activity of Mn-superoxide dismutase and glutathione peroxidase, and depletion of gutathione [40,41]. Peroxynitrite (ONOO^−^) is a particularly powerful oxidant, which results from a diffusion-controlled reaction of NO with O_2_·^−^. In a model of CLP, Escames *et al.* showed increased activity of the inducible mitochondrial nitric oxide synthase (i-mtNOS) in septic mice, which leads to increased mitochondrial ONOO^−^ levels [42,43]. Increased oxidative stress, impairment in OXPHOS function and a decrease in ATP production were restored by genetic deletion of iNOS (iNOS^−/−^ mice), which also includes the mitochondrial isoform, suggesting a significant role of ONOO^−^ in myocardial mitochondrial dysfunction in sepsis [43]. This argument is further supported by the fact that treatment with melatonin, an inhibitor of iNOS, prevented the impairment of mitochondrial homeostasis after sepsis, restored ATP production and improved survival. Some studies also showed an improvement of cardiac function by pharmacological inhibition or genetic deletion of NOS in septic animal models [37,44]. One of these studies showed that recovery of NO production and content of NO metabolites in mitochondria was associated with partial improvement of left ventricular (LV) developed pressure. A causal relationship between mitochondrial NO production and cardiac dysfunction needs to be considered carefully though, since NO is produced by several NOS isoforms (not only mitochondrial) at different intracellular locations and in different cell types, and since the role of NO in cardiovascular function and regulation of vascular tone is indisputably contributing to cardiac dysfunction in sepsis.

While increased ROS levels in sepsis may origin from ROS production at different cellular sites, the importance of mitochondrial oxidative stress in animal models of sepsis has been underlined by studies demonstrating beneficial effects by treatment with mitochondria-targeted antioxidants. Treatment with α-lipoic acid, an antioxidant that is reduced in the mitochondria to a more active form (dihyrolipoic acid), led to full recovery of cardiac mitochondrial function during endotoxemia [45]. Furthermore, administration of Coenzyme Q10 that was conjugated to triphenylphosphonium (MitoQ), a powerful mitochondria-targeted antioxidant, not only restored mitochondrial function but also ameliorated myocardial dysfunction in mice and rats treated with LPS [46]. Finally, treatment with vitamin E conjugated to triphenylphosphonium (Mito-VitE) in a rat pneumonia-related sepsis model prevented mitochondrial ROS production and ROS-related damage in the heart, with recovery of mitochondrial structure and function [32].

### 3.2. Ca^2+^ Handling and Mitochondrial Permeability Transition

On the basis of the chemiosmotic model of energy transduction, the impermeability of the inner mitochondrial membrane is essential to allow the electron transport chain to pump protons across the inner mitochondrial membrane (IMM) and to build up a proton gradient that can be used by the F_0_F_1_-ATPase to regenerate ATP. Mitochondrial permeability transition is a sudden increase of IMM permeability for solutes with a molecular mass up to 1500 Da, and it is believed that this permeability transition is mediated by a voltage- and Ca^2+^-dependent, cyclosporin A (CsA)-sensitive, high-conductance channel located in the inner mitochondrial membrane termed mitochondrial permeability transition pore (mPTP). The consequence of mPTP opening is loss of Δψ, a decrease in State 3 respiration, mitochondrial swelling and rupture of the outer mitochondrial membrane, ultimately leading to activation of pro-apoptotic pathways and necrotic cell death. Ca^2+^ overload is the primary trigger of mPTP opening, but the sensitivity of the pore is dependent on prevailing conditions, such as oxidative stress, adenine nucleotide depletion, increased inorganic phosphate concentrations and mitochondrial depolarization [47]. Using the LPS-induced endotoxemia model in rats, Hassoun *et al.* observed decreased Ca^2+^ uptake into the sarcoplasmic reticulum and increased Ca^2+^ leak from this organelle, which was associated with increased mitochondrial Ca^2+^ content. Administration of the sarcoplasmic reticulum Ca^2+^ leak inhibitor dantrolene prevented mitochondrial Ca^2+^ overload, and improved mitochondrial dysfunction and cardiac contractility defects, suggesting that mitochondrial Ca^2+^ overload could be an important mechanism contributing to myocardial mitochondrial dysfunction in sepsis [30]. Larche and colleagues reported decreased survival, myocardial dysfunction, decreased State 3 and increased State 4 respiration, and increased opening of the mPTP in CLP-induced sepsis. Inhibition of the mPTP by CsA treatment improved cardiac function, survival, and also attenuated mitochondrial dysfunction in this study. In addition, the amount of Ca^2+^ required to increase mPTP opening in CLP-treated mice was less than 25% of the Ca^2+^ needed in control animals, indicative of increased sensitivity for Ca^2+^-induced mPTP opening of the mitochondria in CLP-treated mice [48]. In a different study by Fauvel *et al.*, endotoxemic mice showed decreased LV developed pressure in isolated perfused hearts, and this contractile defect was rescued by *in vivo* treatment with CsA. Treatment with CsA also decreased caspase activation and cytochrome c release, suggestive of prevention of mPTP opening [49]. Chopra *et al.* observed severe morphological deformations that could be indicative of pore formation in rats treated with cecal inoculum, accompanied by a mitochondrial Δψ collapse. Restorative effects were observed in both parameters by treatment with 5-hydroxydecanoate (5-HD), a blocker of mitoK_ATP_ (mitochondrial ATP-sensitive K^+^ channel). Moreover, sepsis-related decreases in cardiac output and ejection fraction were recovered after inhibition of the mitoK_ATP_ [28]. The primary function of mitoK_ATP_ channels is thought to be the regulation of mitochondrial volume; mitoK_ATP_ channel opening results in mitochondrial uptake of potassium and associated inorganic phosphates, anions, and water [50], and is believed to sensitize outer membrane permeabilization and rupture in this sepsis model. Thus, it is tempting to speculate that in some models of sepsis, an impairment in cytosolic calcium handling may lead to mitochondrial Ca^2+^ overload, which triggers opening of a hypersensitive mPTP, thereby contributing to mitochondrial and contractile dysfunction. Further evidence is required to test whether activation of mitoK_ATP_ contributes to increased sensitivity for mPTP opening. It remains to be mentioned though that some studies reported no change of mPTP opening using the mitochondrial swelling assay, although Ca^2+^ handling was found to be impaired in isolated cardiomyocytes [51].

### 3.3. Mitochondrial Uncoupling

Mitchell’s chemiosmotic theory of energy transduction states that the free energy generated by electron transport through the OXPHOS chain and ultimately transfer on O_2_ is used to set up an electrochemical H^+^ gradient across the inner mitochondrial membrane. Protons return into the matrix via the F_0_ subunit of the F_0_F_1_-ATPase, thereby regenerating ATP from ADP. Under physiological conditions, some H^+^ return into the matrix but bypass the F_0_F_1_-ATPase (e.g., via uncoupling proteins (UCPs)), resulting in “mitochondrial uncoupling” of ATP synthesis from O_2_ consumption. Physiologic uncoupling is used for heat generation in brown adipose tissue or to decrease mitochondrial ROS production [52]. Pathologic conditions with increased uncoupling include diabetic cardiomyopathy, where increased utilization of long-chain fatty acids results in ROS-induced activation of UCPs, resulting in decreased amounts of ATP per oxygen consumed and therefore impaired cardiac efficiency (cardiac work/myocardial O_2_ consumption) [53,54,55]. Similarly, an increase in UCP3 expression may increase mitochondrial uncoupling and decrease cardiac efficiency in chronically infarcted failing rat hearts [56].

The role of UCPs in sepsis remains controversial. Twelve hours following CLP, Roshon and colleagues found a 35% reduction in cardiac efficiency in isolated perfused hearts, probably due to a decrease in cardiac work and associated with increased UCP2 mRNA levels [57]. Similarly, myocardial mRNA content of both UCP2 and UCP3 were increased in LPS-induced endotoxemia in rats, and increased myocardial mRNA and protein expression of UCP2 was observed in a canine model of endotoxin-induced shock associated with decreased phosphocreatine/ATP ratios [41,58]. Thus, UCP-mediated uncoupling may impair cardiac efficiency under septic conditions. In contrast, a very recent study reported that treatment of rat embryonic cardiomyoblasts (H9C2) with LPS plus peptidoglycan G led to increased mRNA expression of UCP2, associated with decreased membrane potential, decreased ATP content, increased ROS and depletion of mtDNA. These effects were aggravated by additional silencing of UCP2, suggesting that increased UCP2 expression may actually exert protective effects [59]. Less and also controversial evidence is available on UCP3. Aguirre *et al.* showed increased myocardial UCP3 levels in LPS-induced endotoxemia [60]. These authors report decreased State 3 respiration and Complex IV activity in wildtype and UCP3^−/−^ mice treated with LPS. Proton conductance was found unchanged after 24 h of LPS treatment, regardless of the higher levels of UCP3. In contrast, UCP3 levels were increased with a concomitant loss of membrane potential and ATP content in neonatal cardiomyocytes treated with LPS [61].

### 3.4. Mitochondrial Biogenesis

Mitochondrial biogenesis can be described as growth and division of mitochondria that requires the proper functioning of a number of cellular processes including the coordinated synthesis and import of proteins encoded by the nuclear genome, mtDNA replication, synthesis of proteins encoded by the mitochondrial genome and fusion–fission processes [62]. Mitochondrial biogenesis is triggered by different cellular stressors, such as increased levels of NO, oxidative stress, and an elevated AMP/ATP ratio, among others, resulting in the activation of the peroxisome proliferator-activated receptor y (PPARγ) coactivator (PGC) family of transcriptional coactivators, most importantly PGC-1α and PGC-1β [63,64]. PGC-1α coactivates and/or increases the expression of transcription factors, including ERRα/γ, NRF1/2 and Tfam, that mediate the transcription of nuclear and mitochondrially encoded OXPHOS subunits, nuclear proteins necessary for mtDNA transcription and replication, OXPHOS assembly factors, and components of mitochondrial protein import [65]. By regulating ERRα and PPARα, PGC-1α/β also stimulate the expression of genes encoding for proteins and enzymes of fatty acid oxidation, the main metabolic pathway of fuel oxidation in cardiac tissue.

Several studies have illustrated that mitochondrial biogenesis or the process of generating new mitochondria improves survival in sepsis, and inhibition of mitochondrial biogenesis worsens outcome [25,66]. It is generally thought that loss and/or removal of damaged mitochondria in septic organs is compensated by generation of new mitochondria by increased mitochondrial biogenesis, which should be responsible for increasing survival and reversing organ damage. In the heart, Vanasco *et al.* recently reported increased myocardial levels of PGC1-α and Tfam, accompanied by increased mitochondrial mass and recovery of initially decreased amounts of mtDNA 24 h following LPS treatment [14]. Electron microscopy revealed an increased amount of structurally damaged mitochondria, including swelling, loss and/or disruption of cristae, cleared matrix, and internal vesicles. Despite evidence of mitochondrial biogenesis, mitochondrial State 3 respiration related to Complex I substrates and activity of Complexes I and II were impaired. Others also reported increased expression of mediators of mitochondrial biogenesis, including PGC-1α, NRF-1, and NRF-2, as well as Tfam and the mitochondrial DNA polymerase, associated with mitochondrial structural changes such as swelling and loss of cristae [41]. In neonatal cardiomyocytes, LPS treatment stimulated an increase in Tfam, nuclear accumulation of NRF-1, and expression of PGC-1α, and the changes correlated with signs of mitochondrial dysfunction (decreased mitochondrial membrane potential and ATP content) and markers of autophagy [61]. Taken together, it appears that mitochondrial biogenesis signaling is activated in endotoxemia, which however may not necessarily result in improvement or recovery of mitochondrial function. Russell and colleagues demonstrated that cardiomyocyte-specific overexpression of PGC-1α resulted in a markedly increased mitochondrial biogenesis but also heart failure [67]. Possibly, intensive stimulation of mitochondrial biogenesis might have perturbed the complex process of gene transcription and mitochondrial dynamics in this study, resulting in mitochondria with altered biochemical composition and stoichiometry and thus functional deficits. Thus, while being a compensatory mechanism to overcome loss of dysfunctional mitochondria, uncontrolled triggering of mitochondrial biogenesis in endotoxemia may also result in dysfunctional mitochondria *per se*. Alternatively, mitochondrial biogenesis may simply not be sufficient to compensate for mitochondrial defects in septic conditions.

In contrast to increased mitochondrial biogenesis signaling, Schilling *et al.* showed that LPS injection led to a rapid downregulation of PGC-1α and PGC-1β mRNA levels, accompanied by decreased levels of PPARα, ERRα, and NRF1. Cardiac function was depressed 6 h after the onset of endotoxemia, associated with a decrease in palmitate oxidation in the isolated working heart and increased myocyte lipid accumulation [68]. The total mtDNA copy number and mRNA levels of several enzymes were decreased, and the content of respiratory Complexes I and IV, assessed by 2D-gel electrophoresis and MALDI-TOF mass spectrometry, was reduced in treated rats [41]. Thus, time points and models investigated may affect initiation of mitochondrial biogenesis, and it remains controversial whether activation of mitochondrial biogenesis is actually adaptive or maladaptive under septic and endotoxemic conditions. Of interest, cells overexpressing PGC-1β and mice overexpressing PGC-1β selectively in cardiomyocytes were resistant to the LPS-induced decrease of the expression of PPARα, ERRα, and enzymes of fatty acid oxidation. PGC-1β transgenic mice showed improvement in palmitate oxidation rates in isolated working hearts after treatment with LPS, and echocardiography revealed only a modest decrease in cardiac function [68]. These data suggests that impairment in mitochondrial energetics may importantly contribute to cardiac dysfunction, and that activation of mitochondrial biogenesis may have positive effects in endotoxemia.

When assessing the role of PPARα in endotoxemia, Drosatos *et al.* found that LPS administration reduced the mRNA expression of PGC-1α/β, PPARα, and metabolic PPARα downstream targets. Treatment with a PPARα agonist, however, did not improve cardiac function or alter the mRNA levels of these enzymes after endotoxemia. Interestingly, treatment with rosiglitazone, a PPARγ agonist, led to an improvement of cardiac dysfunction together with improved expression of PGC-1 molecules and Tfam. Moreover, αMHC-PPARγ mice that express PPARγ constitutively in cardiomyocytes showed no disturbances in cardiac function and lipid accumulation after LPS treatment [16]. The exact mechanism by which PPARγ agonism improves or maintains cardiac function in endotoxemia, and in how far these mechanisms are related to mitochondrial biogenesis, remains to be determined.

### 3.5. Mitophagy

Recovery of organ function in sepsis is dependent on removal of dysfunctional mitochondria and generation of new, functionally competent mitochondria, as may be achieved by stimulation of mitochondrial biogenesis. Removal of dysfunctional mitochondria occurs via autophagy of mitochondria, or simply “mitophagy”, which is a quality control mechanism by which cells eliminate dysfunctional mitochondria [69]. Damaged mitochondria are selectively sequestered in autophagosomes and ultimately degraded following fusion with lysosomes. Different pathways have been described to recognize and eliminate the dysfunctional mitochondria. Activation of the PINK1/Parkin (PARK2) pathway leads to ubiquitinylation of special substrates of mitochondria that can be recognized by the adaptor protein p62, which in turn binds to microtubule-associated protein 1A/1B-light chain 3 (LC3). An ubiquitin-independent pathway involves direct binding of autophagy-related protein 8 (ATG8) family proteins to autophagy receptors, such as NIX (Nip3-like protein X) [63,69]. Mitophagy may serve to eliminate dysfunctional mitochondria before they induce cellular damage or cell death, but may also serve as a cell survival pathway by suppressing apoptosis or as a back-up mechanism when the process of apoptosis is defective [70].

Several studies have shown that autophagy and mitophagy are increased in various organs in sepsis [71,72]. In the heart, several studies observed increased myocardial mRNA or protein levels of ATG3, ATG5, ATG7, LC3 II and p62 in LPS-induced endotoxemia, suggestive of increased rates of autophagy [14,16,73]. In some cases, a reduced number of cardiomyocyte mitochondria was reported in LPS-treated animals [16,25,41] potentially suggesting increased mitochondrial degradation by mitophagy, although unchanged mitochondrial mass was observed in other studies [41,74]. An interesting study was published by Piquereau *et al.* who addressed the role of Parkin/PARK2 in mitochondrial dysfunction in endotoxemia. LPS treatment led to a 50% decrease in cardiac output and stroke volume in wildtype and PARK2^−/−^ mice. While wildtype mice completely recovered cardiac function after 48 h, cardiac function remained impaired in PARK2^−/−^ mice. Similarly, a decrease in OXPHOS complex activities, mitochondrial O_2_ consumption rates, and increased susceptibility to mPTP opening were normalized 48 h after endotoxemia induction in wildtype but not PARK2^−/−^ mice. Thus, PARK2-dependent autophagy/mitophagy is required to recover mitochondrial and cardiac function following LPS treatment. In mitochondrial fractions of LPS-treated wildtype mice, levels of polyubiquitinated proteins and PARK2 were increased, and proteins involved in mitochondrial quality control and autophagy (SQTSTM1, LC3 II, BNIP3L/NIX, and BNIP3) were recruited to mitochondria both in wildtype and PARK2^−/−^ mice, showing that septic mitochondria can be targeted for degradation by mitophagy, even in the absence of PARK2. Using electron microscopy, the presence of autophagosome-like structures was confirmed as well [31]. Thus, mitophagy may be increased in endotoxemia and may serve a compensatory role in the recovery of mitochondrial function by eliminating defective mitochondria. The occurrence of mitochondrial biogenesis after the early activation of mitophagy during the course of endotoxemia suggests that an increase in mitochondrial biogenesis may serve to recover an appropriate volume density of mitochondria to restore the full mitochondrial energy-producing capacity of the heart [14,75,76]. Recent studies suggest that the degradation of mitochondria via mitophagy promotes mitochondrial biogenesis directly via a Toll-like receptor 9 (TLR9)-dependent signaling, although the mechanistic links between autophagy and mitochondrial biogenesis are not well characterized yet [72]. In contrast to a beneficial role of autophagy, the controlled elimination of organelles may also be part of apoptosis. Interactions among signaling components of the two pathways (autophagy and apoptosis) have been reported and indicate a complex cross-talk [77]. Thus, it remains unclear whether autophagy is actually an attempt to remove damaged mitochondria or part of a cellular program leading to apoptosis during sepsis. More studies are needed to further define the role of mitophagy in septic cardiomyopathy and to define how mitophagy may directly regulate mitochondrial biogenesis.

## 4. Conclusions

Cardiac dysfunction is common in patients suffering from severe sepsis and septic shock. At present, treatment mainly includes management of the infectious focus and hemodynamic support. However, recent progress in sepsis research increased our understanding of underlying molecular alterations contributing to organ dysfunction, including mitochondrial dysfunction. In the heart, a number of mechanisms have been proposed to underlie myocardial mitochondrial dysfunction, including excessive production of mitochondrial NO and ROS, increased mPTP opening, and increased mitochondrial uncoupling. Several reports showed recovery of mitochondrial function which was associated with improvement or normalization of cardiac pump function following treatment with drugs that target different mechanisms within mitochondria but that had no influence on the inflammatory response, supporting the idea that impaired myocardial energetics rather than cardiac inflammation *per se* are critical for cardiac dysfunction in sepsis [11,23,63]. We now need more studies using pharmacological modulators to prevent or reverse specific mitochondrial mechanisms to further evaluate the significance of mitochondrial mechanisms in septic cardiomyopathy, and to test whether such treatments may be a promising and feasible therapeutic approach that could potentially be transferred from bench to bedside. Such approaches may, for example, include inhibition of mitochondrial ROS and NO production and inhibition of mPTP opening. In addition, it would be very informative to perform such studies in the different models of sepsis and endotoxemia, which may not only reveal differences in the underlying disease mechanisms but may also allow evaluating whether the treatment may be broadly applicable and effective irrespective of the sepsis model. The increasing interest in sepsis research and the availability of novel research tools promises that effective specific treatments may be available in the future to complement our current treatment options of severe sepsis and septic shock to improve outcome for this highly jeopardized patient cohort.
